# Control of cell behaviour through nanovibrational stimulation: nanokicking

**DOI:** 10.1098/rsta.2017.0290

**Published:** 2018-04-16

**Authors:** Shaun N. Robertson, Paul Campsie, Peter G. Childs, Fiona Madsen, Hannah Donnelly, Fiona L. Henriquez, William G. Mackay, Manuel Salmerón-Sánchez, Monica P. Tsimbouri, Craig Williams, Matthew J. Dalby, Stuart Reid

**Affiliations:** 1 SUPA, Department of Biomedical Engineering, University of Strathclyde, Graham Hills, 50 George Street, Glasgow G1 1QE, UK; 2 Division of Biomedical Engineering, School of Engineering, University of Glasgow, Glasgow G12 8QQ, UK; 3 Centre for Cell Engineering, Institute for Molecular, Cell and Systems Biology, College of Medical, Veterinary and Life Sciences, University of Glasgow, Glasgow G12 8QQ, UK; 4 Institute of Healthcare, Policy and Practice, School of Health, Nursing and Midwifery, University of the West of Scotland, Paisley PA1 2BE, UK; 5 Institute of Biomedical and Environmental Health Research, School of Science and Sport, University of the West of Scotland, Paisley PA1 2BE, UK

**Keywords:** nanovibrational stimulation, mesenchymal stem cells, mechanotransduction, nanokicking, bacteria, gravitational waves

## Abstract

Mechanical signals are ubiquitous in our everyday life and the process of converting these mechanical signals into a biological signalling response is known as mechanotransduction. Our understanding of mechanotransduction, and its contribution to vital cellular responses, is a rapidly expanding field of research involving complex processes that are still not clearly understood. The use of mechanical vibration as a stimulus of mechanotransduction, including variation of frequency and amplitude, allows an alternative method to control specific cell behaviour without chemical stimulation (e.g. growth factors). Chemical-independent control of cell behaviour could be highly advantageous for fields including drug discovery and clinical tissue engineering. In this review, a novel technique is described based on nanoscale sinusoidal vibration. Using finite-element analysis in conjunction with laser interferometry, techniques that are used within the field of gravitational wave detection, optimization of apparatus design and calibration of vibration application have been performed. We further discuss the application of nanovibrational stimulation, or ‘nanokicking’, to eukaryotic and prokaryotic cells including the differentiation of mesenchymal stem cells towards an osteoblast cell lineage. Mechanotransductive mechanisms are discussed including mediation through the Rho-A kinase signalling pathway. Optimization of this technique was first performed in two-dimensional culture using a simple vibration platform with an optimal frequency and amplitude of 1 kHz and 22 nm. A novel bioreactor was developed to scale up cell production, with recent research demonstrating that mesenchymal stem cell differentiation can be efficiently triggered in soft gel constructs. This important step provides first evidence that clinically relevant (three-dimensional) volumes of osteoblasts can be produced for the purpose of bone grafting, without complex scaffolds and/or chemical induction. Initial findings have shown that nanovibrational stimulation can also reduce biofilm formation in a number of clinically relevant bacteria. This demonstrates additional utility of the bioreactor to investigate mechanotransduction in other fields of research.

This article is part of a discussion meeting issue ‘The promises of gravitational-wave astronomy’.

## 1. Introduction

The ability for cells to sense and respond to their environment is vital for correct function and ultimately cell survival. The classical view of this process is rooted in terms of chemical signalling, as exploited through biological assays and molecular methods to elucidate signalling pathways. There has been an underappreciation of the importance that mechanical cues play in how cells sense their local environment and trigger signalling (termed mechanotransduction) [1]. This has been a focus of research in recent decades and it is now known that eukaryotic cells have evolved to respond to a plethora of mechanical stimuli (both internal and external to the cell) and physical cues experienced in daily life [2].

The ability to convert mechanical signals into a biological response is recognized as an important mechanism in many species; in humans, it provides the abilities of proprioception, touch, hearing and balance [3,4]. Classically, the view was held that signalling events were primarily controlled through biochemical processes, e.g. enzyme activity and reaction rates. However, there is growing evidence that the physical micro- and nano-environment is critical for the correct functioning and survival of many eukaryotic cells. This is best demonstrated in the absence of mechanical stimuli or through alterations of mechanosensitive genes and proteins. Such mutations have been implicated in the pathology of a number of disease states such as atherosclerosis [5], deafness [6], pathobiology of bones [7], muscular dystrophy [8] and tumours [9]. For example, tumour tissue can be partially diagnosed by assessing increased tissue stiffness, and evidence of changed mechanotransduction response at the single-cell level. The mechanotransductive response of cancer cells is type-dependent, with both reduction of the mechanotransduction response [10,11] and activation of mechanotransduction pathways that promote tumour progression [12]. Changes in tissue stiffness are also implicated in fibrotic lung disease and the inability to respond to the local microenvironment [13].

An underpinning example of the mechanotransductive mechanism is the ability of the mammalian cell cytoskeleton to respond to local physical cues, such as the rigidity of the microenvironment. Exogenous forces are transmitted to cells via the local environment stiffness. The elastic modulus of a material gives an indication of its stiffness, with stiff materials having a high elastic modulus. The human body has a distinct range of elastic moduli from fat at 0.5–1 kPa (soft) through to bone (hard) at 15–20 GPa [14]. Mammalian cells can also sense the force generated by fluid shear stress as demonstrated in flow-cell models [15–17] and force exerted due to gravity [18]. These brief examples illustrate the diverse nature of forces both internal and external that cells sense and respond to. Unravelling the complex mechanisms of mechanotransduction has been aided by the invention of instrumentation and methods able to deliver external mechanical stimuli to individual or multiple cells in culture, for example atomic force microscopy [19] and optical tweezers [20].

This review article provides an overview of mechanotransductive mechanisms based on current experimental studies. Nanoscale vibration is taken as an example to illustrate mechanotransductive pathways along with the associated impact on mesenchymal stem cell (MSC) osteogenesis and bacterial biofilm formation. The key importance of precision measurement and computer-aided design optimization is highlighted in the development of the bespoke nanovibrational bioreactor system used by these studies. Consistency of vibration, being critical to biological reproducibility, is achieved through use of these techniques.

## 2. Cellular response to mechanical stimulus

The sensitivity of cells to their mechanical environment relies on three processes. Firstly, there must be a source of applied force, either externally applied (e.g. hydrostatic pressure, shear flow, gravity) or applied by the cell itself through cytoskeletal contractility. This force must then impose on specific, mechanically sensitive proteins such as extracellular matrix (ECM)-binding proteins, and stretch-sensitive ion channels (e.g. through deformation of the membrane) or the cytoskeleton as a whole. Many proteins are capable of conformational changes or protein folding in response to typical forces imposed by the cellular environment [2]. Conformational change allows new phosphorylation reactions to occur or ion/protein influx in the case of channel proteins, leading to initiation of intracellular signalling [21,22]. Finally, altered signalling can yield changes in cell behaviour following signal transmission to the nucleus. Signal propagation methods include molecular translocation, diffusion and even stress wave propagation directly to the nucleus through the cytoskeletal network [23]. Of these three mechanisms, stress wave propagation can occur rapidly, within timescales of around 1 ms [23], via protein transducers such as membrane-spanning focal adhesion complexes connected to integrins [24]. Studies comparing chemical and mechanical stimulation have demonstrated that the latter can yield significantly faster activation of specific kinases (greater than 12 s versus less than 300 ms, respectively) and this co-localized with areas of cytoskeleton deformation [25].

Focal adhesions demonstrate a key example of tension-based conformational change, with each complex made of multiple subunits forming the overall mechanotransducer. Actin-linking and polymerizing proteins include proteins such as vinculin and talin which connect the cytoskeleton to ECM-bound integrins; however, it is the signalling subunit within the focal adhesion complex that provides force-based modulation of actin polymerization and the actin/myosin contractility process [26]. Proteins such as focal adhesion kinase mediate this signalling by allowing additional phosphorylation to occur following mechanical conformational change leading to downstream signalling [27].

Integrins provide the anchorage which is key to producing increased cytoskeletal tension. Mechanical manipulation of integrins using microbeads demonstrates the entire system in action with axial rearrangement of the cytoskeleton and deformation of the nucleus [28], both effects not seen when manipulating other membrane-bound proteins [29]. Of course, there is a family of integrins with a high degree of specificity in their connection to the cytoskeleton. For example, studies have linked integrin α3β1 with actin filaments and integrin α6β4 with intermediate filaments [30].

Another example of force-based protein manipulation is stretch-activated ion channels, with a number of channels being gated specifically by mechanical force [31]. Opening of these channels creates an ion gradient flow [32,33] which causes diffusion into the cytoplasm and thus possible interaction with various biochemical pathways. In *Escherichia coli* mini, small and large mechanically gated channels play a role adaption to osmosis [33,34]. There are numerous examples of this in eukaryotic cells, including sensory neurons converting the sense of touch to action potentials [35] and in aortic endothelial cells transient receptor potential (TRP) channels are involved with modulation of Ca^2+^ influx when cells are under tension [36]. The specific mechanism of this has been probed through techniques such as patch clamping; however, there is still debate if the gating process is reliant on membrane tension from underlying cytoskeletal proteins (e.g. spectrin) [37] or whether lipid bilayer tension alone is sufficient to activate these channels [38].

Completing the structural picture, it is also important to consider the direct mechanical integration of the ECM to the nucleus via linker of nucleoskeleton and cytoskeleton (LINC) complexes [39]. In a similar manner to the cellular membrane, stress can be directly transmitted to the nuclear envelope, resident ion channels [40] and even the chromatin itself with force-based changes in conformation being possible [41,42]. This was analytically calculated for nanoscale vibration by Curtis *et al.* [43].

**Figure 1.**
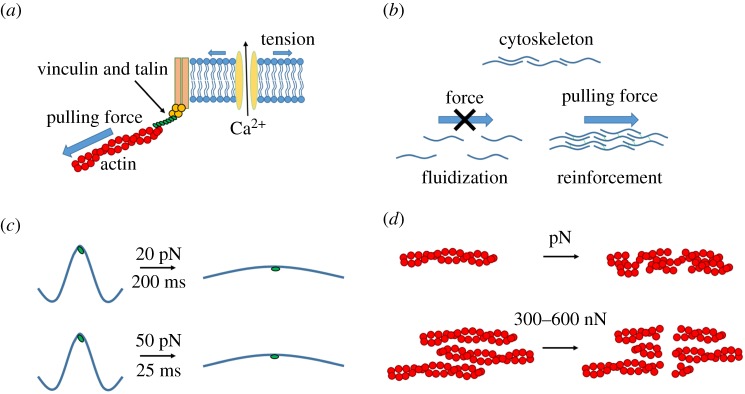
Simplified schematics of switch-like and dynamic elements of mechanotransduction. (*a*) Schematic representation of a common mechanism of mechanotransduction. Conformational change due to tension exerted on a membrane results in the opening of ion channels. This allows a flow of ions across the membrane which triggers specific signalling pathways. The mechanotransduction response is dynamic with factors such as frequency, static versus cyclic stretch and duration of application influencing dynamic mechanotransduction. (*b*) Transmission of force can be directed through the cellular cytoskeleton by a combination of fluidization (stop transmission) or reinforcement (enhance transmission) [44]. (*c*) The magnitude of force applied to a protein filament has been shown to dictate the rate of conformational change. Forces and rates shown were experimentally derived by del Rio *et al.* [45]. (*d*) Reinforced structures are more resistant to breaking; reinforcement of the actin structures, for example, can increase the breaking force by a factor of up to 10 000 [46,47]. The transmission of a force along the structure can also aid in the reinforcement of the structure in a positive feedback loop. (Online version in colour.)

These processes can be summarized using two models: the *switch-like model* and the *dynamic model* [21]. The switch-like model is a basic system which describes the progression of a mechanical signal and how the cell senses and responds to it. This system is summarized in figure 1*a* and shows the process of transmission (mechanotransmission) of the mechanical cue, followed by the sensing of this cue by the cell (mechanosensing) resulting in a biological response (mechanoresponse). Mechanotransmission describes the transmission of the force from adhesion proteins through the cytoskeleton structures, e.g. actin [48], microfilaments [49], microtubules [50] and intermediate filaments [51]. These structures allow forces to travel away from the initial exertion point and propagate along the cell cytoskeleton. As a consequence of the propagation of the force, mechanosensing occurs due to protein conformational changes. It is also important to note that mechanoresponse can describe rapid downstream molecular pathways but may also play a role in long-term response, e.g. arterial wall thickening and bone remodelling [5]. Microgravity is a prime example of altered bone remodelling which has been shown to induce osteopenia [52,53], a loss of bone density.

The switch-like model of mechanotransduction adequately describes the basic molecular diffusion processes in mechanotransduction; however, a dynamic model is required to describe the mechanosensation in force wave propagation as the switch-like model is not appropriate as it fails to account for time-dependent application of force, e.g. continuous stretch versus cyclic stretch [54,55], and the frequency of the stimulus [56]. Specific frequencies have also been observed to elicit different cellular responses. The hierarchical mechanotransduction response of switch-like models forms a basis to understanding the process, but it is apparent that dynamic elements need to be accounted for.

Dynamic mechanotransmission details the transmission of force through the cytoskeleton of the cell, taking into consideration the constant remodelling of the load-bearing structures and the interactions between those different components, e.g. actin–myosin bonds. These adaptive properties of the cytoskeleton can alter local viscoelasticity due to reinforcement of certain structures or fluidization of others (figure 1*b*) [57]. Reinforcement of the cytoskeleton is classically noted in MSC-derived osteoblasts with increased actin stiffening and contractility of the cell when cultured on nanotopographies [58], in response to stiff materials [59], and if spreading is induced through increased cell-adhesion ligation. By contrast, fluidization results in a disruption of the cytoskeletal structures in specific areas. The combination of reinforcement and fluidization thereby allows continued transmission of these forces in a directed manner, where transmission will occur along the reinforced network, but, upon reaching regions where fluidization has occurred, will be dampened [44].

There is strong evidence to suggest that the cytoskeleton can act as a bandgap filter, meaning that the cell is only responsive to certain frequencies [60]. The cytoskeleton is composed of linker molecules that have different elastic constants; altering the cellular ratio of these linkers may allow the ‘filter’ to be tuneable [61]. This is further evidenced by force propagation over longer intracellular distances being controlled by the contractile prestress of the cytoskeleton and the loading frequency [62]. Experimental evidence also suggests that these frequencies are species-specific as shown in the auditory transduction of mammals (rat) occurring faster than that of reptiles (turtle) due to the larger auditory frequency range of mammals [63]. This suggests that dynamic mechanosensing is strongly interlinked with dynamic mechanotransmission when considering the transmission of the mechanical force along the load-bearing structures. As noted, these load-bearing structures are governed by the ratio of the bonds between them and these bonds are also susceptible to being broken when transmitting a mechanical force. Experimental data suggest that forces in the order of hundreds of piconewtons can break single actin fibres, with fibre bundles requiring much larger breaking forces of 300–600 nanonewtons [46,47] (figure 1*c*). Once a bond is broken, transmission of the mechanical force through these two previously bonded structures halts. Dynamic mechanotransduction can also be influenced by the downstream mechanoresponse of the biological pathways involved with the regulation of the cytoskeleton and adhesion structures, effectively acting as a ‘feedback’ loop to enhance or diminish mechanotransduction (figure 1*d*).

## 3. Measurement at the nanoscale

Producing accurate mechanical signals that are transmitted to the cells can be achieved using piezoelectric materials or piezo actuators. Applying a constant electric field to a piezo ceramic deforms its shape; therefore, applying a time-varying electric field, such as a sine wave, can create a source of vibration. In nanovibrational studies, a well-defined sine wave is chosen, allowing the peak acceleration, and therefore the peak force, experienced by the cells to be estimated [43]. Other waveforms (e.g. square waves) have not been studied due to uncertainty in the acceleration and force associated with the corners of the waveform.

Initially, the nanovibrational studies were carried out in small Petri dishes attached to single piezo actuators [43,64,65]. However, the process was scaled up to apply vibration across standard multiwell and flask culturewares [66,67]. The bioreactor design currently consists of an array of piezo actuators sandwiched between a heavy aluminium base and a top plate. The top plate comprises an aluminium and a magnetic stainless steel plate, configured such that the aluminium side is against the array of piezos and the stainless steel plate sits on top allowing cultureware to be magnetically attached.

**Figure 2.**
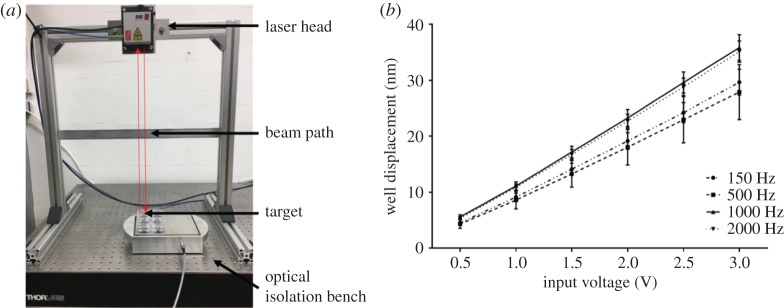
Precision measurement of nanoscale displacements. (*a*) Measurement of nanoscale displacements are performed using laser interferometry. (*b*) Calibration of six-well tissue culture plates on the bioreactor shows a linear correlation between bioreactor input voltage and vibration displacements generated across a range of frequencies. (Online version in colour.)

Nanoscale displacements produced by the bioreactor are measured using laser interferometry, which is used in a diverse range of research areas, including gravitational wave astronomy, where it has been used to make the first direct detection of gravitational waves [68–71]. Gravitational wave interferometers are able to measure displacements of the order 10^−20^ m over a distance of 4 km [68]. In the biological studies reviewed in this paper, a table-top laser interferometer (Model ST-S 120, SIOS Meßtechnik GmbH, Ilmenau, Germany) is used (figure 2), and is capable of resolving displacements of 0.1 nm. Displacement amplitudes across different cultureware types are presented in table 1. Estimation of the accelerative force applied to single cells has previously been calculated using assumptions of the fluid mass being moved during vibration (the mass being accelerated) and found to be in the nN range [43].

**Table 1.** Measurement of vibration displacement produced in a range of commonly used tissue cultureware at 1 kHz. Measurements were performed for each well of the 6- and 12-well plates and at 9 and 21 points on the bottom surface of the T75 and T150 flasks, respectively. Data are mean ± s.d.

	average displacement (nm)	s.d. (nm)
6-well	29.6	±1.9
12-well	31.2	±2.3
T75 flask	31.8	±2.0
T150 flask	33.2	±2.6

**Figure 3.**
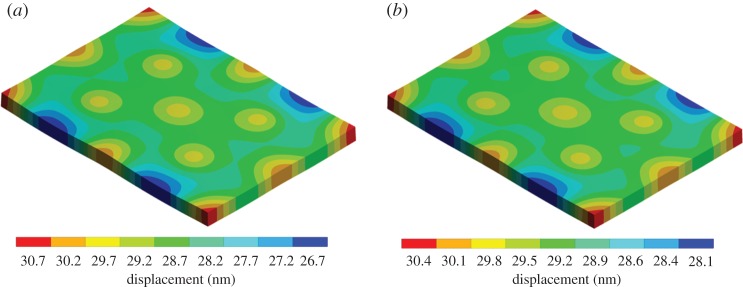
Harmonic analysis of the bioreactor top plate. Modelling of the harmonic response of the bioreactor top plate can be performed to better understand the bioreactor behaviour. (*a*) Harmonic response of the top plate at 1 kHz frequency. (*b*) Harmonic response of the top plate at 2 kHz. Initial 30 nm displacement applied to the underside of the top plate to simulate the action of the 13 piezos. To simulate gravitational loading, an acceleration of 9.806 m s^−1^ was applied in the opposite direction of piezo action. (Online version in colour.)

Finite-element analysis (FEA) modelling has been critical in the design of the bioreactor to predict and correct for the effect of vibrational resonance in the device and cultureware. A modal analysis can provide information on exactly how the chosen cultureware will deform at each of its resonant modes. For example, it was shown in [65] that a 52 mm Petri dish has a resonant mode at 339 Hz when mechanically stimulated by a single piezo at the centre of its base. The saddle shape of this mode would result in cells near the edge of the dish receiving almost double the vibration amplitude than the cells at the centre of the dish, leading to inconsistent cell stimulation. This analysis highlights that all cultureware will have an upper frequency limit for the production of consistently stimulated cells which could be significant not only for research-based experiments, but also for future medical trials where reproducibility and consistency are crucial. Suitable modification of the cultureware (increasing rigidity) can help increase the first internal resonance and thus extend the frequency range where the system behaves as a rigid body. In addition, harmonic analysis can be used to inform the design of the bioreactor and cultureware, e.g. placement of piezos and material selection, predicting the nanoscale displacements transmitted to the cultureware and thus cells. An example of the bioreactor top plate is shown in figure 3, where a subtle difference in the predicted nanoscale displacements is shown when comparing a 1 and 2 kHz frequency.

## 4. Mesenchymal stem cell differentiation and mechanical stimulation

Bone is the second most transplanted tissue in humans and is commonly grafted from the iliac crest (donor site) to the recipient site [72]. The volume of autologous bone which can be removed this way is limited and can often be associated with chronic donor-site pain, post-operative infection and other donor-site morbidities [73]. Bone undergoes modelling and remodelling in response to physical external factors to maintain structural strength and mineral homeostasis. This remodelling is controlled through the actions of osteoblasts and osteoclasts, bone-building and bone-resorbing cells, respectively. Imbalances in the endosteal resorption of bone and periosteal apposition may lead to conditions such as osteoporosis [74]. MSCs are multipotent stromal cells that differentiate into a number of cell types associated with the musculoskeletal system such as osteoblasts, adipocytes, chondrocytes and fibroblasts [75]. MSCs reside in virtually all postnatal tissue [76,77] but are commonly isolated from the bone marrow [78]. Adipose (fat) tissue is also rich in adipose-derived MSCs [79].

Controlling MSC differentiation is, therefore, highly desirable to address the clinical need, as such experimental techniques have focused on passive (e.g. topographical control and environmental stiffness) and active methods (e.g. gravity, shear flow and vibration). Passive techniques focus on the generation of internal tension in the cell by altering the physical environment the cells attach to. This can be accomplished by altering the material or by topographical patterns, whereby different levels of stress can be achieved by varying the parameters [80]. Microscale patterns are a well-researched area, and effects have been shown to have a marked effect on cell behaviour and ultimately, in the case of MSCs, differentiation. These effects can also occur through alteration of the surface with specific ECM proteins and/or polymers [81–83]. Addition of these components to the surface or changing the electrostatic potential of the surface can alter the absorbed ECM protein [84]. Microscale features have been extensively investigated aided by relevant technology, e.g. photolithography, and have been found to regulate cell functions including but not limited to proliferation, differentiation and apoptosis [82–84]. With the advance of microfabrication techniques, extensive investigation of ordered microscale structures, e.g. pillars [85,86], pits [87] and groves [88,89], was achievable and contributed greatly to the effect these structures have on cellular mechanotransduction. Microscale features in the context of implants have been noted to alter osteoblast behaviour [90,91].

Nanoscale features known as nanotopographies have been shown to have a strong impact on the morphology and phenotype of MSCs [92,93], and to alter other cell responses such as proliferation. Dalby *et al.* [92] used a series of ordered and disordered nanoscale grids formed from pits with geometry 120 nm diameter and 100 nm deep. While the ordered nanoscale grids showed minimal osteogenesis, the disordered near-square pattern showed the largest increase compared to the hexagonal and perfect square patterns. A further study, altering the height of titania nanopillars, demonstrated an inverse relationship between osteoinductive effect and feature height (15 nm being optimal) [94]. Reduction of the nanopillars to 8 nm diminished this effect, suggesting that there was a critical cut-off size for cell filopodia interaction with nanofeatures. These experiments also revealed fine nanoscale projections, promoted by the 8 nm features, which are now termed ‘nanopodia’. Additional investigation revealed large changes in adhesion, nucleus and lamin morphologies, leading to the suggestion that direct (mechanical) and indirect (biochemical) signalling are critically important in regulating stem cell fate [95]. It has also been shown that 350 nm gratings affect human MSC adhesion and migration [93]. It was noted that mature focal adhesions of a smaller size were observed, and zyxin was identified as being responsible for this due to reduced intracellular tension. It was hypothesized that the 350 nm gratings showed a similar response to compliant surfaces. Thus, how MSCs adhere and spread on materials is important for subsequent differentiation. Surfaces that stimulate adhesion drive increased cytoskeletal contraction and enhanced osteogenesis [96,97]. A classical example is from confinement of MSCs to small adhesive areas (e.g. 1000 µm^2^), which restricts cell spreading and thus induces adipogenesis, while larger surface areas (e.g. 10 000 µm^2^), which facilitates cell spreading, induces differentiation of MSCs towards an osteoblast lineage. This occurs due to changes in actin–myosin contraction mediated through Rho-A kinase (ROCK) [44].

In the human body the local environment in which cells reside can cover a diverse stiffness range from <kPa through to tens of GPa [14]. Mechanotransduction processes can be activated by prestress in the cytoskeleton in addition to the potential that alteration of the rigidity may also be sufficient to have an impact on cellular differentiation.

MSCs have been shown to respond to the elasticity of their environment and stiffness gradients, e.g. between tissue types. Hydrogels are a valuable tool for the investigation of cellular response within a “3D” matrix. These gels can be synthesized from an array of biological and synthetic polymers with the ability to modulate the elastic modulus by changing the cross-linking [98,99]. The ability to tailor stiffness coupled with being highly hydrated has resulted in hydrogels being useful in mimicking human tissue to study cell behaviour [100,101]. Mimicking the elasticity of three distinct tissue types, that of brain, muscle and bone, hydrogels seeded with MSCs showed phenotypic switches towards the cell type of the mimicked tissue type [59]. Another study demonstrated that MSCs are capable of migrating along these gradients (1 to 100 Pa µm^−1^) to the stiffest regions of these gradients in a process known as durotaxis [102]. In addition, their data suggested that a functional actin cytoskeleton is required to achieve this migration, and microtubules are required for this migration to be directed.

Active methods such as gravity, compressive loading and shear have also been shown to be inducers of MSC differentiation. Hypergravity (10 g) has been shown to increase cell proliferation in addition to upregulation of runt-related transcription factor (RUNX2), which gives some indication of an increase in osteoblastogenesis [103]. Cyclic compressive loading has been shown to have the potential to induce chondrogenesis of rabbit bone marrow-derived MSCs by synthesis of TGF-β1 [104]. Combination of cyclic compressive loading with added TGF-β1 showed no significant difference when compared with TGF-β1 and cyclic compressive loading only. Although shear flow is often thought of as influencing endothelial cells due to their periodic cycling of pressure and flow when exposed to blood flowing in the human body [105], there is also evidence to suggest that low interstitial shear flow can induce MSCs towards an osteogenic lineage. Low sheer flow stress induces osteogenesis of MSCs by activating the transcriptional coactivator with PDZ-binding motif (TAZ), which activates the TAZ target genes CTGF and Cyr61 [106].

## 5. Controlling mesenchymal stem cell behaviour using nanovibrational stimulation

Vibration is classed as an active technique and is viewed as being a cyclically compressive force. Owing to the dynamic nature of the cellular cytoskeleton and its adaptive nature to external stimuli, vibration has been used to investigate mechanotransduction at the cellular level. The experimental apparatus used has ranged from cultureware attached to speakers [107], horizontal vibration [108] and culture plate shakers [109]. A study of periodontal ligament stem cells using low-magnitude, high-frequency vibration (LMHF) over a frequency range of 10–180 Hz observed promotion of osteogenic differentiation with the optimal frequency being 50 Hz [109]. A study of MSCs combining shear flow (range 0.04 to 5 Pa) and a vibration of magnitudes 0.15, 1 and 2 g with frequencies of 30 and 100 Hz demonstrated a commitment to an osteogenic lineage [110]. Furthermore, this commitment was due to an upregulation of the actin-remodelling genes including the Wiskott–Aldrich syndrome (WAS). This mechanism was found to be independent of shear flow. Intermittent vibrational loading of hASCs over a 3 day period using a square wave of 50 Hz and 100 Hz with a maximum acceleration magnitude of 3 g resulted in an increase of some osteogenic markers but failed to approach that of the osteogenic media [107]. In contrast, stimulation of adipose-derived stem cells (AT-MSCs) using subsonic vibration at a frequency range of 10–40 Hz resulted in differentiation of AT-MSCs towards a neural lineage [111]. Owing to the diverse range of apparatus used in vibrational experiments, it is difficult to compare and contrast studies. In addition, it becomes difficult to determine the factor(s) responsible for the observed biological responses when amplitudes are not measured or stated in studies. It may also suggest that differentiation is sensitive to acceleration or that specific frequencies and/or amplitudes play an important role in differentiation driven by mechanotransduction.

It is also of interest that vibration has potential clinical applications, in particular the application of low-magnitude, high-frequency (LMHF) vibration, also known as whole-body vibration (WBV). These signals have been found to elicit an anabolic effect in murine bone models [112,113]. WBV has been implicated clinically as being effective in intervention in lower back pain and potential therapeutic effectiveness for sarcopenia and osteoporosis [114]. Safe exposure to WBV is important as long-term WBV has been shown to negatively affect certain individuals in industrial settings with lower back pain and sciatica [115].

**Figure 4.**
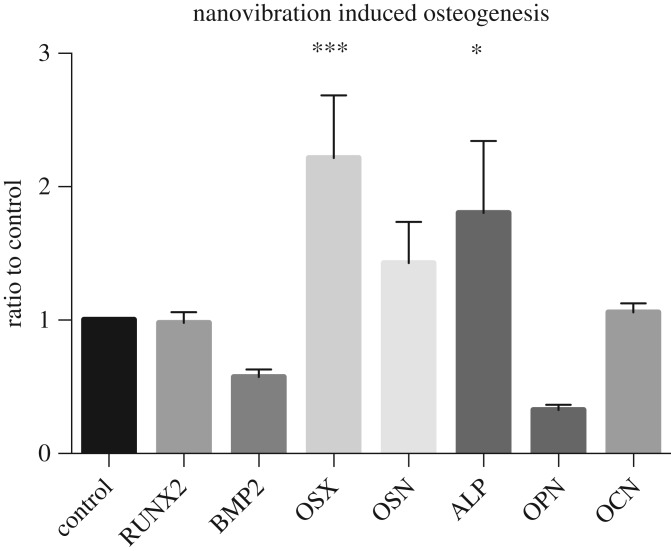
Nanovibrational stimulation induces osteogenesis of MSCs. Osteogenic induction of MSCs by nanovibrational stimulation (1 kHz, 22 nm displacement) after 7 days in 2D culture. Osteogenic gene expression was measured by qRT-PCR for RUNX2 (Runt-related transcription factor 2), BMP2 (bone morphogenetic protein 2), OSX (Osterix), OSN (Osteonectin), ALP (alkaline phosphatase), OPN (Osteopontin) and OCN (Osteocalcin). OSX and ALP were statistically significantly higher in the nanostimulated samples in comparison to the unstimulated control (error bars = s.d., 1 patient, *n* = 4, one-way ANOVA ***p < 0.005).

**Figure 5.**
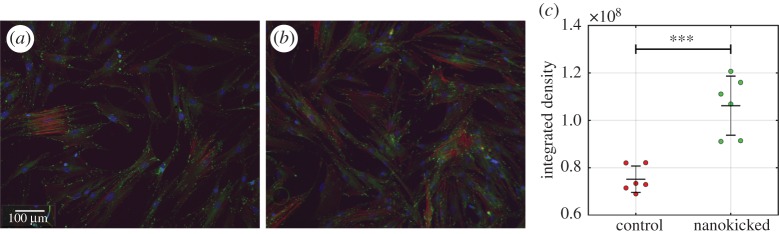
Enhanced adhesion and cytoskeletal remodelling in response to nanovibrational stimulation. Nanovibrational stimulation was applied to MSCs (1 kHz, 22 nm displacement). Fluorescence labelling of actin cytoskeleton (red), vinculin (green) and cell nucleus (blue) was performed. Representative images of (*a*) control and (*b*) nanovibrational stimulation applied to MSCs, showing induced remodelling of cell cytoskeleton and increased focal adhesion formation of the stimulated cells. Scale bar, 100 µm. (*c*) Integrated density measurements of vinculin expression. Increased vinculin expression in NK (1 kHz) samples in comparison to non-stimulated controls. Data are mean ± s.d., Student *t*-test, *p* < 0.001. *n* = 6. (Online version in colour.)

As a cell interacts with surfaces, nanoscale membrane undulations occur that influence cell-surface interaction [116]. These vibrations produced by the cell have specific amplitudes, frequencies and timescales depending on the cell type [116–122]. Given that cell membranes naturally vibrate at the nanoscale, Curtis and colleagues investigated the application of nanovibrational stimulation using piezo actuators [43]. Osteogenic stimulation of MSCs was noted through activation of the Rho-kinase (ROCK) pathway (figure 4), where significant upregulation of osterix and alkaline phosphatase was measured at day 7 [65]. Osterix is an osteoblast-specific transcription factor and a major effector in bone formation [123]. *In vitro* induction of alkaline phosphate (ALP) has previously been shown as a robust predictor of bone-forming capacity *in vivo*; however, this only occurred when alkaline phosphatase, ALP mRNA levels and ALP activity were observed during *in vitro* osteogenic differentiation [124]. MSCs have demonstrated a strong morphological change in response to nanovibrational stimulation when grown on Petri dishes as monolayer, two-dimensional (2D) cultures (figure 5). This occurs due to increased contractility of the cytoskeleton and actin reorganization. An increase in vinculin, a focal adhesion protein linking integrins to F-actin, is also observed in response to nanovibrational stimulation [125].

Nanovibrational stimulation at 500 and 1000 Hz was also shown to influence the size of the nucleus of the cell. The peak force experienced per cell was estimated to be nanonewtons in magnitude [64]. A combination of nanovibrational stimulation with nanotopographies has also been investigated and showed some benefit of having both environmental and mechanical stimulation; however, nanovibrational stimulation was found to provide a stronger osteoblastic cue [126].

**Figure 6.**
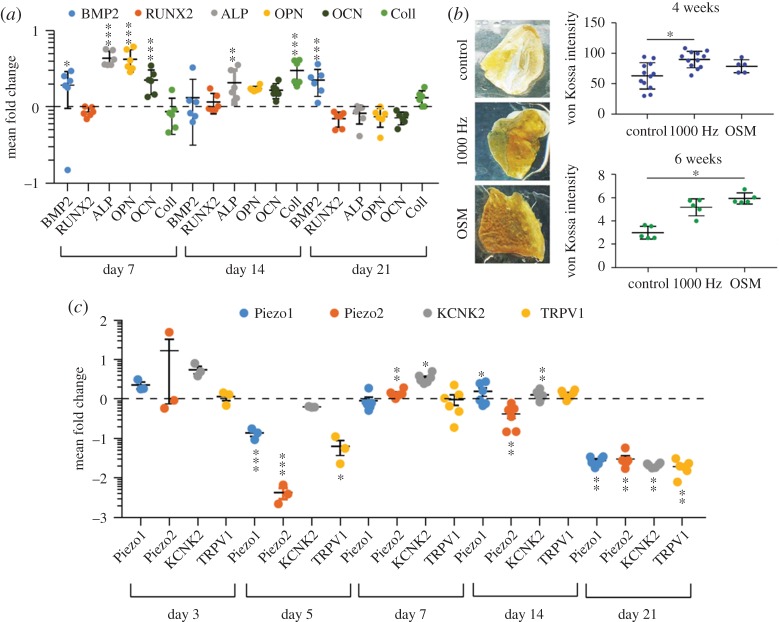
Nanovibrational stimulation induces osteogenesis. (*a*) qRT-PCR analysis of osteogenic markers over time relative to no vibration. The expression of BMP2, ALP, OPN and OCN was significantly higher in 3D nanovibrated MSCs compared to control. Donors (D) = 1, replicates per donor (r) = 6, technical replicates per replicate (t) = 2; results are mean ± s.d., statistics by Mann–Whitney *U*-test where *p < 0.05, **p < 0.01 and ***p < 0.001. (*b*) Osteogenesis measurement by Von Kossa staining. Staining was performed after 6 weeks of stimulation at 1 kHz in 3D. Representative gels shown (on the left) were removed from a six-well plate prior to staining. A significantly increased level of staining was observed for the nanostimulated samples relative to the unstimulated controls after 4 weeks (D = 3 (D = 1 for OSM), r = 4), and for nanostimulated samples and osteospecific media (OSM). OSM-treated cells after 6 weeks (D = 1, r = 5). Results are mean ± s.d., *p < 0.05 by ANOVA and the Kruskal–Wallis test. (*c*) Temporal qRT-PCR data for Piezo1, Piezo2, TRPV1 and KCNK2 transcripts in MSCs after 3, 5, 7, 14 and 21 days of 3D nanovibrational stimulation displayed as the mean fold change with respect to no-vibration control cultures. A trend can be observed: high to low expression, followed by recovery, and by another phase of high to low expression, with significant downregulation of all the receptor transcripts at day 21. Donors (D) = 1, replicates per donor (r) = 6 (3 for days 3 and 5), technical replicates per replicate (t) = 2; results are mean ± s.d., statistics by Mann–Whitney *U*-test, *p < 0.05, **p < 0.01 and ***p < 0.001. Values are displayed in log scale. Adapted from Tsimbouri *et al.* [67]. (Online version in colour.)

*In vitro*, cells are routinely cultured in two dimensions; however, this has been found to be a poor representation of the cellular response *in vivo*, where interactions occur in three dimensions with the ECM alongside a host of bioactive factors [127]. The ECM is composed of macromolecules, glycosaminoglycans and fibrous proteins, of which collagen and fibronectin are components [128]. A study was performed to investigate if nanovibrational stimulation could be applied to a 3D hydrogel structure using the bioreactor discussed in §3 [67]. A low elastic modulus scaffold (collagen) was used because high elastic modulus scaffolds alone have been shown to induce osteogenesis of MSCs. FEA modelling and interferometry confirmed transmission of vibration from the nanovibrational bioreactor to the scaffold at the desired 1 kHz frequency. Significant upregulation of BMP2, ALP, OPN and OCN at day 7 and significant upregulation of ALP and collagen I at day 14 was observed (figure 6*a*). The upregulation of these genes, relative to the control, is strongly indicative of osteogenesis. At day 21, downregulation of RUNX2, ALP, OCN and OPN was observed, which indicated that the transcriptional component of osteogenesis had completed. OCN protein expression at day 21 further confirmed the completion of the transcriptional component of osteogenesis. Mineralization was confirmed using a combination of von Kossa staining for phosphate, Raman spectroscopy and X-ray micro-computed tomography. Mineralization was higher than the control and, at 4 weeks of culture, was higher than that observed in osteoinductive media (figure 6*b*). Collectively, these results provide strong evidence that nanovibrational stimulation provides a strong osteogenic cue in a non-osteogenic 3D environment. To further elucidate the mechanotransduction mechanisms by which osteogenesis was induced, ion channel sensitivity was tested with the mechanoreceptors: Piezo, TRPV1 and KCNK being differentially expressed in a temporal manner (figure 6*c*). The involvement of these mechanoreceptors supports a role of intracellular tension in osteogenesis-induced mechanotransduction.

**Figure 7.**
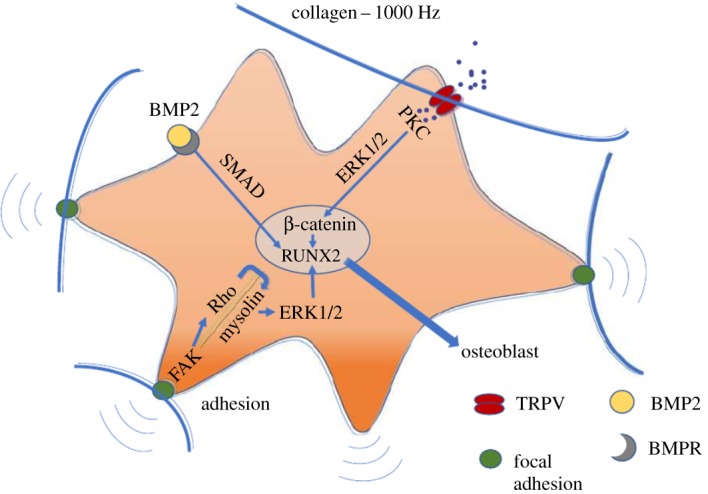
Proposed mechanotransduction mechanism. Schematic representation of proposed pathways involved in the regulation of osteogenesis in response to nanovibrational stimulation. The TRP-β-catenin pathway is highlighted as it has been experimentally validated as being important to the MSC nanovibrational-induced osteogenesis. Adapted from Tsimbouri *et al.* [67]. (Online version in colour.)

Metabolomics analysis further revealed that TRPV1 is the major contributor to Wnt-mediated osteogenesis, being involved in the activation of protein kinase C and ERK-mediated β-catenin activity. A hypothesis for the proposed mechanisms involved in nanovibrational mechanotransduction is summarized schematically (figure 7).

## 6. Applying nanovibrational stimulation to prokaryotic cells

Mechanotransduction is deemed essential to the normal function of many mammalian cells, while in contrast this mechanism is rarely considered for bacteria where chemical signals are often regarded as dominant [129–131]. Bacteria, however, routinely experience mechanical forces in flow systems, cell-to-cell interaction, cell-to-surface interaction, twitching motility and the change from planktonic to sessile (biofilm) growth mode. Vibration has been observed in Gram-positive bacteria in a species-dependent manner with ranges from 21 nm (*Staphylococcus epidermidis* ATCC35984) up to 145 nm (*Streptococcus salivarius* HB7) [132]. The switch-like model can be applied to bacteria but may suffer the same limitations as found with mammalian cells as bacteria experience a diverse range of mechanical forces. Bacteria have been found experimentally to have mechanosensitive channels, specifically *E. coli* which has three classes of mechanosensitive channels in varying size (mscL, mscM, mscS) [133]. There is, however, no direct evidence that these channels respond to external mechanical force due to the limited number of studies of mechanotransduction in bacteria. Evidence of mechanotransduction in bacteria has been shown in *Pseudomonas aeruginosa* (*P. aeruginosa*) where type IV pili (tfp) have been identified as part of a mechanotransduction system, implicated in modulating surface attachment [134]. Retraction of the pilus directly induces signal transduction of a chemotaxis sensory system known as Chp. The Chp system in turn regulates cyclic adenosine monophosphate production and genes associated with virulence. *Neisseria gonorrhoeae* (*N. gonorrhoeae*) tfp have also been demonstrated to play a significant role during infection, stimulating microcolony formation and cytoprotection [135,136]. During infection, *N. gonorrhoeae* tfp and epithelial cells have also been observed to be involved in physical cross-talk and hijacking of the epithelial cell's mechanotransduction mechanism [137].

Other mechanical forces have been demonstrated to influence bacterial phenotype, particularly shear flow. The production of extracellular polymeric substances has been shown to increase biofilm cohesion under shear flow [138]. High-velocity conditions (tending to and reaching turbulent flow) resulted in thinner biofilms with a greater total amount of polysaccharides and proteins, in addition to decreased attachment compared to biofilms grown under low-velocity conditions. External application of force via vibration to a surface has been performed using low-energy surface acoustic waves (SAWs) to induce displacements [139]. SAWs were shown to significantly reduce microbial biofilm formation of *Candia albicans*, *E. coli*, *Proteus mirabilis* and *Escherichia faecalis* in Foley catheters. The actuators typically generated vibrations that were in the 100 to 300 kHz range with amplitudes of 300 to 800 nm at source, resulting in SAWs between the 0.2 and 2 nm displacement. Another study has shown that vibration produced by an acoustic method (speaker with Petri dish attached to the speaker surface) enhanced biofilm formation of *P. aeruginosa* and *Staphylococcus aureus* (*S. aureus*), using frequencies of 800 and 1600 Hz with displacements of roughly 100 nm [140]. Non-uniform displacement was evidenced by the presence of standing waves in the media, clustering of latex beads and biofilm striation in concentric rings out from the centre of the Petri dish, indicators of multiple force vectors.

**Figure 8.**
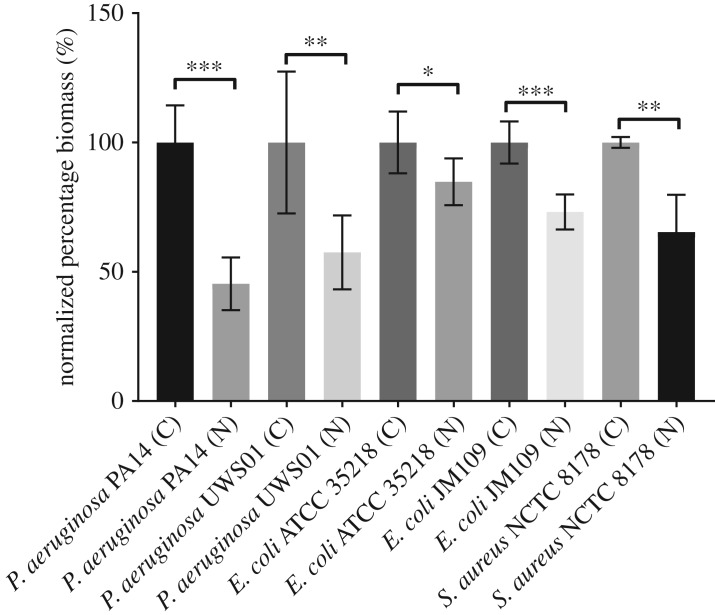
Reduction of bacterial biomass under nanovibrational stimulation. A selection of clinically relevant bacteria were stimulated (1 kHz, 30 nm) for 24 h, using a set-up nominally identical to that used in the initial MSC nanovibrational studies [65]. Crystal violet staining to quantify total biomass was performed at the end point. (C) = Control, (N) = nanovibrational stimulation. Data normalized to control = mean ± s.d., Student *t*-test, * *p* < 0.05, *** *p* < 0.001 *** *p* < 0.0001. *n* = 3.

It is apparent that mechanotransduction is vitally important for microbes to sense and respond to their external environment. Our understanding of microbial mechanotransduction is relatively poor in comparison to that of mammalian cells, yet understanding these mechanisms will provide insight and potential of modulating or controlling bacterial behaviour. Nanovibrational stimulation, due to the precision and ability to produce uniform displacements, may prove useful in bacterial mechanotransduction studies. Preliminary studies of the biofilm formation capacity of a number of clinically relevant bacterial species have been performed using the same apparatus used in the initial MSC nanokicking study [65]. Vibration with a frequency of 1 kHz and displacement of 30 nm was applied immediately upon inoculation of bacteria over a period of 24 h. Significant reduction in the overall biofilm formation was observed with *P. aeruginosa* PA14, *P. aeruginosa* UWS01, *E. coli* ATCC 35218, *E. coli* JM109 and *S. aureus* NCTC 8178 compared to the control (figure 8) using a total biomass crystal violet staining method. This gives tentative first evidence that nanovibrational stimulation may find application in the control of bacterial biofilm formation of both Gram-positive and Gram-negative bacterial species. It is as yet unknown how bacteria respond at a molecular level to this nanoscale stimulation; research is ongoing to evaluate this response. It may also be of interest to compare the molecular mechanisms involved in bacterial response with those involved with eukaryotic cellular response, to determine whether there is a conserved evolutionary mechanism in the response to mechanical forces.

## 7. Conclusion

Nanovibrational stimulation of cell cultures can be applied using the novel bioreactor platform described within this paper. This system has been developed through computer simulations (FEA) and validated using laser interferometry, both exploited within the field of gravitational wave astronomy. The bioreactor described in this work has significant advantages compared to previous systems, particularly in relation to the compatibility with standard cultureware, and in the supply of mineralizing osteoblasts in a 3D matrix. In addition, as the bioreactor does not come into contact with the mineralizing matrix, adaption towards a good manufacturing practice process is straightforward and regulatory approval for ancillary reagents is not required.

The role of mechanotransduction and how mechanical signals influence cell behaviour are receiving increased attention by researchers and clinicians. The use of nanoscale vibration (nanokicking) has been successfully exploited in promoting osteogenesis from mesenchymal stem cells, both in 2D and 3D constructs. This paves a novel route to fabricate tissue-engineered bone graft for regenerative medicine, for use in the repair of non-union bone fractures. As noted, other cell types, including two clinically relevant forms of bacteria, have been reported to respond to nanoscale vibration, suggesting that the scope of this technique is significantly broader than simply controlling MSC differentiation.

The exploitation of this technique to date has arisen from multidisciplinary research between gravitational wave physicists and biologists. This combination of expertise continues to explore the wider biological potential of this technique in a research and clinical environment as well as developing new technologies.

## Data accessibility

The raw data are held within the repositories provided in the original publications. New raw data generated for this review are held within http://dx.doi.org/10.5525/gla.researchdata.592.
